# Molecular adaptation of *Lactobacillus plantarum* WCFS1 to gallic acid revealed by genome-scale transcriptomic signature and physiological analysis

**DOI:** 10.1186/s12934-015-0345-y

**Published:** 2015-10-09

**Authors:** Inés Reverón, Blanca de las Rivas, Ruth Matesanz, Rosario Muñoz, Félix López de Felipe

**Affiliations:** Laboratorio de Biotecnología Bacteriana, Instituto de Ciencia y Tecnología de los Alimentos y Nutrición (ICTAN-CSIC), Madrid, Spain; Centro de Investigaciones Biológicas (CIB-CSIC), Madrid, Spain

**Keywords:** Transcriptomics, Gallic acid, *Lactobacillus plantarum*, Pyrogallol

## Abstract

**Background:**

Gallic acid (GA) is a model hydroxybenzoic acid that occurs esterified in the lignocellulosic biomass of higher plants. GA displays relevant biological activities including anticancer properties. Owing to its antimicrobial and cellulase-inhibiting activities, GA also imposes constraints to the fermentability of lignocellulosic hydrolysates. In depth-knowledge of the mechanisms used by tolerant microorganisms to adapt to hydroxybenzoic acids would be a step forward to improve the bioavailability of GA or select/engineer production hosts with improved metabolic traits for the bioconversion of pretreated lignocellulosic biomass.

**Results:**

Whole genome transcriptional profiling using DNA microarrays was used to characterize the molecular response of *Lactobacillus plantarum* WCFS1 to GA. Expression levels of 14 and 40 genes were differentially regulated at 1.5 and 15 mM GA, respectively. The transcriptomic analysis identified a marked induction of genes with confirmed or related roles to gastrointestinal survival, the repression of genes coding for certain ABC-type transporters and modulation of genes involved in the control of intracellular ammonia levels, among other responses. Most notably, a core set of genes dedicated to produce GA from polyphenols (*tanB*_*Lp*_), decarboxylate GA to pyrogallol (*lpdB*, *lpdC* and *lpdD*) and transport functions (*lp_2943*) was highly overexpressed at both GA concentrations. Correspondingly, resting cells of strain WCFS1 induced by GA, but not their non-induced controls, produced pyrogallol. Gene expression and organization of genes involved in GA metabolism suggested a chemiosmotic mechanism of energy generation. Resting cells of *L. plantarum* induced by GA generated a membrane potential and a pH gradient across the membrane immediately upon addition of GA. Altogether, transcriptome profiling correlated with physiological observations indicating that a proton motive force could be generated during GA metabolism as a result of electrogenic GA uptake coupled with proton consumption by the intracellular gallate decarboxylase.

**Conclusions:**

The combination of transcriptome and physiological analyses revealed versatile molecular mechanisms involved in the adaptation of *L. plantarum* to GA. These data provide a platform to improve the survival of *Lactobacillus* in the gut. Our data may also guide the selection/engineering of microorganisms that better tolerate phenolic inhibitors present in pretreated lignocellulosic feedstocks.

**Electronic supplementary material:**

The online version of this article (doi:10.1186/s12934-015-0345-y) contains supplementary material, which is available to authorized users.

## Background

Edible plants are the main sources of dietary phenolic acids, particularly hydroxycinnamic and hydroxybenzoic acids, which are micronutrients with significant biological properties. Gallic acid (GA) is a model hydroxybenzoic acid widely distributed in edible plants and occurs in several legumes, fruits, vegetables, nuts and beverages of plant origin [1]. GA displays relevant biological activities including anti-inflammatory [2] or antiviral ones [3] and has attracted interest because of its reported anticancer properties in animal and in vitro studies (reviewed in [4]). In the context of symbiosis with human host, the gut microbiota has evolved biochemical pathways for the bioactivation/degradation of dietary polyphenols [5]. Hence, the bioaccesibility and health effects of GA depend on the activity of the subset of gut microbes that resist the anti-bacterial activity reported for tannins [6] and GA itself [3]. Therefore, a better understanding of the tolerance of gut microbiota to these health-relevant compounds will be a step forward to improve the persistence of beneficial microorganisms in the intestine.

In addition to its significant biological activities, the GA esterified in the lignocellulosic biomass can be released during the pretreatment required for the efficient industrial uses of cellulosic materials by microorganisms [7]. On account of its anti-microbial activity [6] [3], released GA and tannins (precursors of GA) have inhibitory effects on these microorganisms. In addition, these compounds are phenolic inhibitors of enzymes used for cellulose hydrolysis to produce ethanol [8] and consequently impose constraints to the fermentability of lignocellulosic hydrolysates. Therefore, in depth-knowledge of the mechanisms used by microorganisms to overcome the toxicity of hydroxybenzoic acids is of interest to assist in the selection and/or engineering of production hosts with improved metabolic traits for the bioconversion of lignocellulosic biomass.

Lactobacilli are suitable model microorganisms to study resistance mechanisms to phenolic acids as they display higher tolerance to phenolic compounds than other bacterial groups [9]. In fact, some *Lactobacillus* ssp. have been chosen as models to obtain datasets of specific expression profiles in response to model hydroxycinnamic acids such as ferulic [10] and *p*-coumaric acids [11]. Regarding the hydroxybenzoic acids, a proteomic approach revealed some of the molecular adaptive responses to the GA precursor, tannic acid [12]. Among the molecular mechanisms used to persist under tannic acid-stress *Lactobacillus plantarum* relies on tannase (tannin acyl hydrolase) [13], an enzyme that transform the gallate esters of tannins into GA and glucose. Recently, the elusive gallate decarboxylase activity (GDC), which decarboxylates GA to yield pyrogallol (PG) as final product of tannin metabolism, has been uncovered in *L. plantarum* WCFS1 [14]. Despite this crucial advance in the understanding of GA metabolism, knowledge on how gut microorganisms respond to hydroxybenzoic acids is not completely understood.

To provide insight into the microbial mechanisms involved in the tolerance to hydroxybenzoic acids, the current work describes the molecular adaptive responses of the model bacterium *L*. *plantarum* WCFS1 to GA as studied by whole-genome transcription profiling. Based on this transcriptional analysis, several mechanisms involved in the response to GA are proposed. The main response identified by the transcriptional datasets, the GA-inducible catabolism of GA to PG, was corroborated by specific metabolic analysis. The transcriptome-based results and the organization of genes involved in GA decarboxylation pointed towards a chemiosmotic mechanism of energy generation associated to GA metabolism, which was experimentally supported by membrane potential and internal pH measurements.

## Results

### Global transcriptomic responses during adaptation to GA

To investigate the adaptive response of *L*. *plantarum* WCFS1 to GA, the transcriptomic profile of *L. plantarum* WCFS1 was defined in cells exponentially growing in medium devoid of GA after 10 min of exposure to 1.5 or 15 mM of this compound. The time of exposure was chosen considering the short half-life of mRNAs reported for genes involved in stress responses induced by phenolic acids in *L. plantarum* [15]. The concentrations of GA used (1.5 and 15 mM GA) cover a range which could be representative of the amounts of GA present in the diet, provided that an estimated dietary intake of ≈6 mmol (1 g) GA/day has been reported by some authors [16].

The impact of GA on the transcriptomic profile of *L. plantarum* WCFS1 was evaluated by sorting all genes whose transcript level showed changes (log_2_ratio) of at least ±1.5 (*p* < 0.05). Overall, 14 genes were significantly differentially expressed at 1.5 mM GA whereas 40 transcripts were affected at 15 mM GA (15 upregulated and 25 downregulated). The differentially expressed genes, functionally distributed according to their Clusters of Orthologous Groups (COGs) categories, are shown (http://www.ncbi.nlm.nih.gov/COG/ and ftp://ftp.ncbi.nih.gov/pub/COG/COG2014/static/lists/listCOGs.html) (Additional file 1: Table S1). The induction of global stress responses was not detected in the transcriptome analysis. In addition, no downregulation of genes coding for ribosomal proteins or translation factors or associated with transcription was evident (Additional file 1: Table S1), indicating that the stringent response was not triggered by GA.

A core set of genes, including *lpdB* (*lp_0271*), *lpdD* (*lp_0272*), *lpdC* (*lp_2945*), *lp_2943* (ion transporter), *lp_2956* (*tanB*_*Lp*_) (tannase), *lp_2940* (surface protein which has been reportedly demonstrated to play a key role in the persistence and survival of *L. plantarum* WCFS1 in the GI-tract of mice [17]) and *lp_0274* (transcriptional regulator), were highly overexpressed (Additional file 1: Table S1). In addition, the gene *amtB* (NH_4_^+^ transport protein involved in regulation of nitrogen metabolism) was downregulated. These genes were considered as the pivotal response to GA, as their expression showed the same trend and was roughly conserved at both GA concentrations.

Beside this response, other responses involving the carbohydrate and nitrogen metabolisms were observed at 15 mM GA. At this higher GA concentration several *L. plantarum* genes coding for ABC-type transporters were significantly downregulated (see below), whereas only two genes putatively involved in stress response pathways were upregulated (Additional file 1: Table S1). In addition, some regulatory networks were activated. These and other variations in the transcriptomic response of *L. plantarum* to GA are detailed in the following sections.

### Correlation between gene expression, GA metabolism and the generation of a proton motive force

#### GA-mediated induction of genes associated with the transport and metabolism of GA

The GA-responsive genes most strongly upregulated in this study code for LpdC (*lp_2945*) (272.1-fold induction at 15 mM GA), LpdB (*lp_0271*) (26.3-fold induction at 15 mM GA) and LpdD (*lp_0272*) (24.0-fold induction at 15 mM GA). These are the three subunits of the recently uncovered intracellular gallate decarboxylase (GDC) of *L. plantarum* WCFS1 [14] which decarboxylates GA into PG. The second most upregulated gene upon exposure to GA, *lp_2943* (73-fold induction at 15 mM GA), was the sole gene in this study displaying homology with genes coding transporters of the IT (ion transporter) superfamily, which suggest the involvement of the *lp_2943* product in GA transport. This transcript profile strongly suggests that the presence of GA is required to induce the transport of GA and its intracellular decarboxylation to PG.

#### Correlation between GA metabolism and transcriptome profiles

**Fig. 1:**
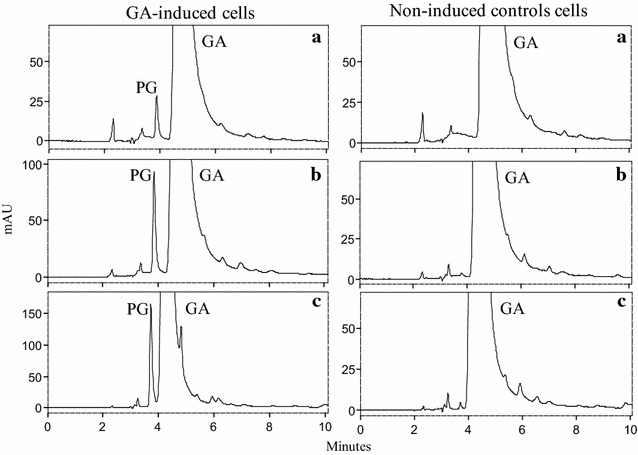
Gallic acid metabolism in resting cells of *L. plantarum*. A concentration of 1.5 mM gallic acid (GA) was added to washed cells of GA-induced *L. plantarum* WCFS1 (*left panel*) or non-induced *L. plantarum* WCFS1 (*right panel*). The GA and PG present in the supernatants after 15 (**a**), 30 (**b**) and 60 (**c**) min of incubation were detected by HPLC analysis

To investigate whether the overexpression of genes associated to GA metabolism correlated with changes in its anticipated phenotype (gallate decarboxylase activity), PG production (end product of GA metabolism) was tracked in resting cells prepared from GA-induced and non-induced cultures. As shown in Fig. 1, only resting cells prepared from GA-induced cultures (left panel) of *L. plantarum*, but not from non-induced cultures (right panel), were able to decarboxylate GA to PG. These PG production patterns reveal a specific transcriptome to phenotype association and validate the correlation between overexpression of genes coding for the GDC (*lpdB, lpdC* and *lpdD*) and its predicted phenotype. Location of the GDC in the cytoplasm [14] and the use of resting cells in the assays, instead of cell-free extracts, show that GA is transported into the cell and PG extruded to the external milieu. The rates of GA conversion and PG production were measured by HPLC and could only be calculated in the experiments using resting cells prepared from GA-induced cultures. The GA was decarboxylated by these cells at a rate of 0.011 mmol l^−1^ min^−1^, which was commensurate with a PG production rate of 0.012 mmol l^−1^ min^−1^. These data show that decarboxylation of GA to PG is stoichiometric, excluding further PG degradation under our conditions.

#### Generation of a proton motive force during GA metabolism

The genetic organization displayed by *lp_2943*, which clusters downstream *lp_2945* (catalytic subunit of the GDC activity [14]), resembled that of some decarboxylation pathways built from a precursor/product exchanger and a decarboxylase that are activated to supply chemiosmotic energy by the generation of a proton motive force (PMF) across the membrane [18]. This observation, besides the strong induction of *lp_2943* and genes coding for the GDC mediated by GA, led us to ask whether a PMF was generated during GA metabolism.

**Fig. 2:**
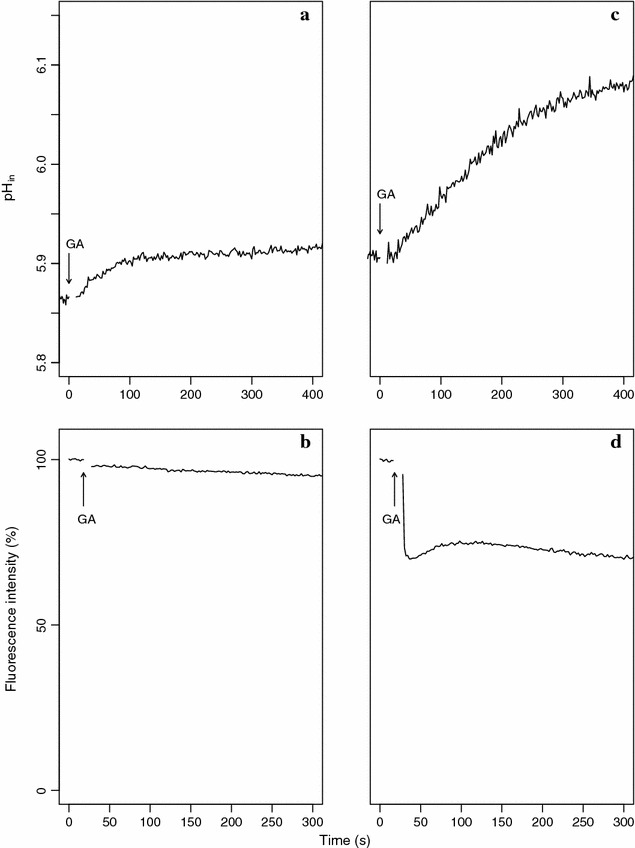
Energetics of gallic acid metabolism in *L. plantarum* WCFS1. The internal pH (**a**, **c**) and the membrane potential (**b**, **d**) of GA-induced cells (**c**, **d**) and non-induced cells (**a**, **b**) were continuously tracked in time. GA (1.5 mM) was added at the times indicated by the *arrows*. Cells were loaded with BCECF as described in “Methods”. Variations in membrane potential were quantitatively evaluated from the quenching of the potentiometric probe DisC_3_

To test this hypothesis, the pH gradient across the membrane (∆pH) and the membrane potential (∆Ψ) (the two components of the PMF), were tracked with the fluorescent pH indicator BCECF and the fluorescent probe DiSC_3_, respectively (Fig. 2). GA-induced and non-induced cells resuspended in 50 mM Kpi pH 5.8 buffer displayed a cytoplasmic pH similar to the external pH (Fig. 2a, c). Addition of 1.5 mM GA to GA-induced cells resulted in alkalinization of the cytoplasm and an increase of the transmembrane pH gradient of 0.2 pH units (Fig. 2c). In contrast, non-induced control cells showed almost no change in internal pH (Fig. 2a).

The membrane potential was qualitatively measured with the fluorescent probe DiSC_3_. Variations in the ∆Ψ showed the same trend as those obtained for the transmembrane pH gradient. Thus, addition of 1.5 mM GA only increased the ∆Ψ in resting cells induced with GA (Fig. 2d) while non-induced resting cells did not generate a ∆Ψ upon the same conditions (Fig. 2b).

The experiments show that *L. plantarum* generates a proton motive force during GA transport and metabolism.

### Effects of GA on central metabolic functions

#### Carbohydrate metabolism

Microarray data showed the upregulation (2.0-fold) of genes encoding for glucosamine-6-phosphate deaminase/isomerase (*nagB*), which is involved in *N*-acetyl glucosamine (GlcNAc) utilization. GA also induced the transcription of *lp_2954* (~3.0-fold) which, according to LocateP (a genome-scale subcellular-location predictor [19]), codes for a hypothetical membrane protein of unknown function. Since the structure of a protein is better conserved than the amino acid sequence during evolution, the putative *lp*_*2954* product was submitted to the Phyre2 modelling server (http://www.sbg.bio.ic.ac.uk/~phyre/) to probe the likely extent of structural similarity to other proteins. This search showed an integral membrane protein EIIC of a PTS system which transports disaccharides with predicted specificity for *N, N´*-diacetylchitobiose ([GlcNAc]_2_), as the best hit of the predicted protein. The *lp_2954* gene coding for this putative PTS transporter lacks the accompanying genes coding for the structural components EIIA and EIIB, suggesting that it is one of the so called “orphan” PTS-EIIC transport components. The coincident upregulation of *nagB* and *lp_2954* suggests that GlcNAc utilization is promoted by the presence of GA.

Genes coding for a ribose transport protein (*lp_3659*) and a ribose kinase (*lp_3660*) were downregulated and a gene coding for a phosphoketolase (*lp_2659*), involved in boosting the carbon flow from the pentose phosphate pathway to glycolytic intermediates, was also underexpressed. Furthermore, *lp_3015* encoding for a lytic transglycosidase that hydrolyzes the glycan strands of the cell wall peptidoglycan was downregulated (3.4-fold).

#### Nitrogen metabolism

Microarray data showed the downregulation of *glnR* (*lp_1580*), which codes for a major transcription factor that acts as the global repressor of nitrogen metabolism in *L. plantarum* [20]. Downregulation of *glnR* was accompanied by the concurrent underexpression of genes encoding functions involved in the assimilation and reallocation of nitrogen within the cell. More particularly, genes coding for functions involved in influx and production of glutamine (Gln) and ammonium, i.e. *glnA* (glutamine synthetase), *lp_0822* (glutamine-fructose-6-phosphate transaminase), genes coding for a Gln ABC transporter (*lp_0802* and *lp_0803*), *lp_2830* (aspartate ammonia-lyase which produces fumarate from Asp with the production of ammonium), *amtB* (NH_4_^+^ transport protein), were all downregulated.

Among the genes involved in nitrogen metabolism, only *glnH* (*lp_2312*) was upregulated. This gene codes for the substrate binding GlnH subunit of a *L. plantarum* putative *glnPQH* ABC transport system. According to Interproscan (http://www.ebi.ac.uk/InterProScan), the putative GlnH protein is predicted to contain a domain with similarity to an ionotropic glutamate receptor, suggesting that this ABC transport system could transport glutamate, which is essential for *L. plantarum* growth.

### GA represses the expression of predicted ABC-type transporters

The adaptive response to GA in *L. plantarum* included the downregulation (2 to 7.2-fold at 15 mM GA) of six clustered genes coding for components of two ABC-type transport systems (*lp_2739*-*lp_2740* and *lp_2743*-*lp_2744*), a putative transcriptional regulator (*lp_2742*) and a small peptide (*lp_2741*). The *lp_2739* and *lp_2743* genes encode the putative ATPases of their respective ABC systems, whereas *lp_2740* and *lp_2744* display transmembrane domains and encode the putative permeases. The ABC transporter encoded by *lp_2739*-*lp_2740* is homologous to the BceAB transport system from *B. subtilis*, which functions as a detoxification pump of peptide antibiotics and antibiotics unrelated to antimicrobial peptides [21].

The predicted *lp_2742* product (123 aa) displays the signatures of YtrA subfamily of the GntR superfamily regulators: a reduced C-terminal domain (which tends to be shorter in this subfamily) with only two α-helices [22]. The regulators of the YtrA subfamily often respond to antibiotic stress [23] and typically modulate the expression of ABC transport systems [22]. Accordingly, the predicted *lp_2743* product is homologous to an ABC component of the YtrA operon from *B. subtilis*, which responds to cell wall antibiotics [23].

### Redox and hydrolase related functions responsive to GA

We noticed the overexpression of two clustered genes, *lp_1424* and *lp_1425*. The gene *lp_1425* encodes for one of the six redundant copies of the fumarate reductase subunit A (FrdA) from *L. plantarum* WCFS1 [24] and *lp_1424* encodes for a putative NADPH-dependent FMN oxidoreductase, both encoding proteins predicted to be located in the cytoplasm [19]. The best Phyre hits of the putative *lp_1424* product were found with members of the NADH_dh2 family of putative flavin-binding quinone reductases. Fittingly, the predicted *lp_1424* product contains the signature sequence LPVTPEYN*XXXXXX*LKNAID*XX*S typical from the members of this family.

Interestingly, the presence of GA induced a marked decrease (4.1-fold) in *lp_2229* transcripts encoding for a protein with homology to hydrolases of the metallo β-lactamase (MBL) superfamily. The predicted *lp_2229* product displays the HXHXDH motif (HYHHDH; residues 59–64) absolutely conserved among all the MBLs [25] and presented the best Phyre hits with Zn-dependent MBL-like hydrolases which use tRNA and mRNA substrates. One of these MBL families, the UlaG family of proteins [26], is functionally specialized to process substrates distinct from nucleic acids.

### Regulatory networks

Some genes involved in the response of *L. plantarum* to GA clustered together or were grouped in operon structures, suggesting co-regulatory relations. Downregulation of *glnR* (*lp_1580*) coding for GlnR, the master regulator of nitrogen metabolism in *L. plantarum*, was concurrent with the downregulation of *glnA* (*lp_1581*), *ansB* (*lp_2830*), *amtB* (*lp_0349*) and a *glnPHQ* glutamine ABC transport system (*lp_0802* and *lp_0803*) fitting with the previously predicted GlnR regulon architecture [20].

Besides GlnR, another GA-responsive regulons of *L. plantarum* were the regulon Lp_2742 (http://regprecise.lbl.gov/RegPrecise/regulon.jsp) encompassing genes *lp_2739* to *lp_2744*, which is predicted to be controlled by the transcription factor encoded by *lp_2742* (GntR family, YtrA subfamily) and the RbsR regulon which encompass *rbsK* (*lp_3660*) and *rbsD* (*lp_3659*) genes.

## Discussion

Here, we have explored the mechanisms of adaptation of *L. plantarum* to GA. Gene expression-wide analyses did not reveal stringent or general stress responses. The most striking response to this model hydroxybenzoic acid, the inducible decarboxylation of GA to PG, was revealed by the combination of transcriptome and physiological analyses.

**Fig. 3:**
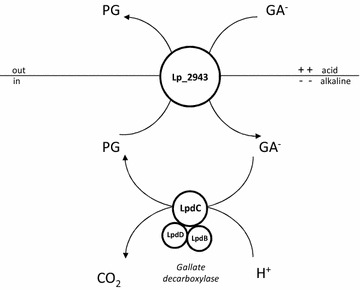
Proposed mechanism of proton motive force generation by gallic acid metabolism in *L. plantarum* WCFS1. *GA* ^*─*^ mono-anionic gallic acid, *PG* pyrogallol

A membrane potential was rapidly generated by *L. plantarum* upon addition of GA, but it was only observed in resting cells obtained from cultures induced with GA. Hence, the observed ∆Ψ could only be associated to a (transport) function induced by GA, given the membrane potential is formed by a transporter during substrate uptake. Since *lp_2943* was the sole gene coding for an ion transporter induced by GA, its pronounced overexpression strongly suggested the *lp_2943* product was the GA transporter. Typically, transporters involved in PMF-generating decarboxylation pathways function as exchangers of a product that is structurally similar to and more positively charged than the precursor [18], as it is the case for GA (single charged anion) and PG (uncharged). Further, our results indicated that PG production is intimately linked to ∆Ψ generation, as both processes could only be observed in resting cells induced by GA. Considering the above, we propose that the observed membrane potential was generated by an electrogenic GA/PG exchange catalysed by the *lp_2943* product which would translocate a net negative charge from the medium to the cytoplasm. Similarly to the membrane potential, the transmembrane pH gradient component of the PMF was only observed in GA-induced cells. This result correlates with the robust induction of the GDC-encoding genes by GA and the GA-inducible nature of PG production. Therefore, these results strongly suggested that the transmembrane pH gradient was generated by proton consumption in the GDC step of the pathway. Altogether, gene organization and transcriptome profiling correlated with physiological data indicating that a PMF was generated by GA decarboxylation and electrogenic transport of GA (most probably in exchange with the decarboxylated product, PG). The proposed PMF-generating system (comprised of *lp_2943* and the GDC-encoding genes) (Fig. 3) can be considered as a new mechanism of energy generation in lactobacilli, which would be analogous to known PMF-generating systems of bacteria consisting of a precursor/product exchanger and a decarboxylase. Some examples of these energy-generating decarboxylation pathways are the malolactic fermentation by some lactic acid bacteria, the decarboxylation pathway of oxalate to formate by *Oxalobacter formigenes* or the decarboxylation of some amino acids to form biogenic amines (for an excellent review on these and other PMF-generating systems see Sobczak and Lolkema [18]).

The marked downregulation of genes coding for certain ABC transporters, and its homology-based function prediction related to the response to cell wall drugs, suggests that they could also be involved in the traffic of GA and PG across the membrane, especially as it has been reported that these transporters are usually stress-responsive proteins extruding a wide range of drugs [27]. A gene (*lp_2229*) coding for a putative MBL-like hydrolase homologous to the UlaG family, which uses tRNA and mRNA substrates, was notably downregulated. The UlaG family is poorly characterized, but it has been suggested that the ancestral RNase fold of this protein had been subjected to extensive modifications in order to acquire new catalytic activities which appear to be very diverse in bacteria [28]. For example, UlaG is a MBL-like hydrolase that has been functionally specialized to act in the cytoplasm as l-ascorbate 6-phosphate lactonase involved in l-ascorbate metabolism [28]. The emergence of these novel metabolic functions, coupled with the fact that LP_2229 is predicted to be located in the cytoplasm (according to LocateP), could provide a rationale for the functional specialization of the *lp_2229* product to hydrolyse the aromatic ring of GA, although this remains to be proven. If these were the functions of the MBL-like hydrolase and the downregulated ABC-transporters, the observed adaptation (downregulation) could be directed to preserve GA availability and integrity in order to foster the PMF-generating effects linked to the GA metabolism mentioned above.

The induction of genes coding for redox related functions, such as *lp_1424* and *lp_1425*, would be expected since GA is a quinol and it tends to autoxidize and/or undergo vicarious reduction by cellular one-electron reducers [29] thus generating highly oxygen reactive semiquinones. According to its intracellular location and predicted functions, the GA-mediated induction of the gene pair *lp_1424*/*lp_1425* could be targeted to activating one-step reduction of intracellular quinols to its corresponding quinone (*lp_1424*) and vice versa (*lp_1425*). If active, this redox cycle would preempt the autoxidation and/or vicarious reduction of quinols that, such as *p*-coumaric and gallic acids, have been transported into the cytoplasm. In support of this hypothesis, the *lp_1424*/*lp_1425* gene pair was also previously shown to be strongly induced by *p*-coumaric acid stress [11].

The transcriptional analysis also identified the differential expression of genes involved in nitrogen metabolism. Beyond the response observed at 1.5 mM GA, which only affected the *amtB* gene, transcriptional changes related to nitrogen metabolism were expanded to genes of the GlnR regulon at a higher GA concentration (15 mM). The concurrent downregulation of *glnR* (coding for the GlnR repressor) and the genes encompassing the GlnR regulon indicates that GlnR-mediated regulation in *L. plantarum* occurs in a similar manner to that of *Bacillus subtilis* [20]. Supporting this hypothesis the *L. plantarum* GlnR and glutamine synthetase (GlnA) encoding genes are organized, like in *B. subtilis*, as *glnRA* operon. In *B. subtilis* GlnR represses the transcription of the *glnRA* operon (negative autoregulation) [30]. In this bacterium the presence of the feed-back inhibited glutamine synthase promotes GlnR dimerization (activation) and increases the affinity of GlnR for the binding site thus acting as a chaperone which stabilizes the GlnR-DNA complexes [31]. The downregulation of genes encompassing the GlnR regulon, fits with the recently proposed role of GlnR: to prevent the influx, and simultaneously limit the intracellular production, of ammonia and glutamine [20]. In addition, the downregulation of genes involved in ribose transport (*lp_3660* (transporter)) and utilization (*lp_3659* (kinase)) is probably related to the tight control of ammonia levels, as ribose catabolism is closely linked to nucleoside metabolism. Thus, purine biosynthesis from ribose 5-P contributes to regenerate 2-ketoglutarate which is interconverted with glutamine and glutamate in central metabolic conversions to assimilate and re-distribute nitrogen within the cell [32]. This tight control of ammonia levels avoids diffusion of ammonia out of the cell and ensures sufficient biosynthetic nitrogen assimilation flux required for growth [33]. The fact that GA act as a signal to accumulate nitrogen could be related with the low nutrient availability encountered in plants, the natural habitat of *L. plantarum*. In fact, ammonium efflux is limited in *L. plantarum* under conditions of extremely low growth mimicking that encountered in plant environments [32].

The insights on the molecular adaptive responses of *L. plantarum* to GA revealed by the transcriptional datasets identified a marked induction of functions dedicated to metabolize tannic acid and GA, two phenolic compounds with antimicrobial activity and potent inhibitory effects towards cellulases [8]. This knowledge may be used to guide the design of improved detoxification pretreatments of cellulosic hydrolysates by selection/engineering of production hosts well suited to remove these fermentation inhibitors.

Interestingly, genes encoding functions involved in the adaptation of *L. plantarum* to the GI-tract conditions, such as *lp_2940* and *lp_2956* (tannase), were highly overexpressed by GA. The crucial role played by *lp_2940* in the persistence and survival of *L. plantarum* WCFS1 in the GI-tract has been already reported [34], albeit the molecular mechanism involved in this function remains to be elucidated. Regarding tannase, it may confer advantages to microbes to persist in the GI-tract as tannins display antimicrobial activity [35] and reduce the species richness in the gut [36]. Other responses to GA related to an increased survival of *L. plantarum* in the GI-tract include the induction of genes involved in GlcNAc utilization, which is part of the human intestinal mucus glycoproteins and can be used as carbon source by *Lactobacillus,* particularly under bile stress [37]. Altogether, these expression profiles indicate that contact with GA could improve the persistence of *L. plantarum* in the GI-tract. This hypothesis is in line with the accumulating evidence that the response of *L. plantarum* to some dietary polyphenol provides crossprotection against gastrointestinal stress [11–13]. Further, the induction by GA of genes required for tannin and GA metabolism provides valuable information to develop strategies aimed at increasing the bioactivity and health effects of these metabolites. For instance, tannase has been recently observed as an underlying function of the microbiota that selectively colonizes colorectal cancer tissues [38]. Considering the anti-carcinogenic roles displayed by GA and PG (end products of tannin metabolism), successful strategies could be designed to kill colorectal cancer cells, more in view of the strong GA-mediated up-regulation of genes required for tannin and GA metabolism observed in this study.

## Conclusions

This report expands our insight into the molecular mechanisms underlying the response of *Lactobacillus* to hydroxybenzoic acids. The transcriptome signature of GA response coincided with physiological analyses to show that the GA catabolic pathway required induction by the substrate (GA), and that the energetic consequence of GA metabolism in *L. plantarum* was the generation of a proton motive force (PMF). Expression profiling at genome scale provided insights on how GA operates globally suggesting that it acts as a signal to trigger versatile responses including nitrogen storage, tannin metabolism or GlcNAc utilization. The ability of *L. plantarum* to synthesize energy from GA in the form of PMF and the GA-mediated induction of nitrogen storage, utilization of carbon sources such as GlcNAc (which is used in the GI-tract under bile stress) or tannins (which exerts antimicrobial properties on intestinal bacteria), potentially confer competitive advantages to this microorganism in the GI-tract where nutrients are not in constant supply. This view is supported by the GA-mediated induction of genes playing a crucial role in the survival in the GI-tract. The strong GA-mediated induction of tannase and the enzymes involved in the GA catabolic pathway, highlight the ability of some lactic acid bacteria to better tolerate phenolic inhibitors that are present in pretreated lignocellulosic feedstocks, and further support the suggested potential of these microorganisms to become cell factories for the efficient conversion of cellulosic biomass [39].

## Methods

### Bacterial strain and culture conditions

*Lactobacillus plantarum* WCFS1 kindly provided by Dr. Michiel Kleerebezem (NIZO Food Research, The Netherlands) was grown in Man-Rogosa-Sharpe (MRS) broth (Difco Laboratories, Madrid, Spain) [40] at 30 °C without shaking. This strain is a colony isolate of *L. plantarum* NCIMB 8826, which was isolated from human saliva. It survives the passage through the human stomach [41] and persists in the digestive tract of mice and humans better than other *Lactobacillus* spp. isolated from the human intestine [42]. GA (Sigma) stock solution (200 mM) was prepared in MRS. Appropriate dilutions were used to adjust GA final concentration to 1.5 or 15 mM in MRS.

### RNA extraction

Twelve paired independent *L. plantarum* WCFS1 batch cultures (50 mL each) were grown in MRS lacking GA to an OD_660_ ≈ 0.8–0.9. Then a culture of each pair (twelve cultures) was induced with GA to bring their final concentration to 1.5 mM or 15 mM (twelve biological replicates per each GA concentration). The induced cells and their corresponding controls were centrifuged at 4 °C after 10 min of exposure to this phenolic acid. The pellet was mixed with 2 mL of quenching buffer (60 % methanol, 66.7 mM HEPES, pH 6.5, −40 °C). Following quenching, the cells were centrifuged at 9000×*g* for 10 min at −10 °C and suspended in an extraction mixture (500 μL 1:4 chloroform-acid phenol, 30 μL of 10 % SDS, 30 μL Na-acetate 3 M pH 5.2, 400 μL Tris–EDTA buffer [10 mM Tris(hydroxymethyl)amino methane, 1 mM EDTA] pH 7.4, 15 mg of polyvinylpoly-pyrrolidone, and 500 mg of glass beads (ϕ, 75–150 μm). The cells were broken under frozen conditions in a FastPrep™ Fp120 (SAVANT) using three treatments of 5000 rpm for 40 s and chilled 1 min between cycles. The suspension was then centrifuged at 4 °C at 10,000×*g* for 2 min. After two extractions with 500 μL of chloroform the supernatant containing the RNA was immediately frozen in liquid nitrogen, and stored at −80 °C [43]. NanoDrop ND1000 instrument was used for RNA quantification. The A_260_/A_280_ and A_260_/A_230_ ratios were measured to check RNA purity. Integrity and quality of RNA samples were determined by electrophoresis on agarose gels. Two treatments with DNase I (Ambion) were applied and the absence of genomic DNA was confirmed by PCR [43].

### Microarray: cDNA synthesis, purification and hybridization

Before first-strand cDNA synthesis, RNA integrity was evaluated using the Agilent 2100 Bioanalyzer (Agilent Technologies). Fluorescently labelled cDNA was obtained by using the SuperScript Indirect cDNA Labeling System (Invitrogen). After, the Cy3 and HyPer5 fluorescent dyes (Amersham Biosciences) were coupled to the aminoallyl-modified first-strand cDNA, and purification of probes was carried out with the CyScribe GFX Purification Kit. Labeling efficiency was assessed using a NanoDrop ND1000 spectrophotometer. Preparation of probes and hybridization at 65 °C during 17 h was performed as described on the Two-Color Microarray Based Gene Expression Analysis Manual (Quick Amp Labeling) with Tecan HS Pro Hybridization (V. 5.7/Agilent Technologies). Slide *L. plantarum* WCFS1 8x15 K microarray GE Agilent G2509F Oligo Microarrays (No. 026636) was custom designed and contains 60-mer probes that were taken at the gene expression omnibus database (GEO Accession No.GPL5874). The oligo-microarray contained an average of three probes per transcript.

### Real-time quantitative RT-PCR assays (qRT-PCR)

Real-time qRT-PCR was used to validate the microarray data. Amplification was carried out using a 7500 Fast System (Applied Biosystems). RNA was reverse transcribed using High Capacity cDNA Reverse Transcription Kits (Applied Biosystems). The specific primers used for the qRT-PCR assays are listed (see Additional file 2: Table S2). The SYBR Green method was used and each assay was performed in triplicate using SYBR Green real-time PCR Master Mix (Applied Biosystems). Amplification was initiated at 95 °C for 10 min, followed by 40 cycles of 95 °C for 15 s and 60 °C for 1 min. Control PCRs were included to confirm the absence of primer dimer formation (no-template control), and to verify that there was no DNA contamination (without RT enzyme negative control). All real-time PCR assays amplified a single product as determined by melting curve analysis and by electrophoresis. A standard curve was plotted with cycle threshold (Ct) values obtained from amplification of known quantities of cDNAs and used to determine the efficiency (E) as E = 10^−1/slope^. The expression levels of target genes were normalized. The Bestkeeper analysis [44] was applied, and the geometric mean of the most stably expressed housekeeping genes (16S rRNA*, ldhD, gapB, dnaG,* and *gyrA*) was used as a normalization factor. The expression ratios measured by microarrays and by qRT-PCR assay were plotted, and the linear correlation coefficient was calculated (y = 0.973x + 0.107; R^2^ = 0.97) (see Additional file 3: Table S3).

### Data analysis

Images were captured with a GenePix 4000B (Axon) and spots quantified using GenPix software (Axon). Background correction and normalization of expression data were performed using the methods normexp and loess in LIMMA, respectively [45]. The expected False Discovery Rate (FDR) was controlled to be less than 5 %. Genes were considered differentially expressed when nominal *p* values were <0.05 and had a fold change (FC) equal or higher than ±1.5. FC was calculated as the average of the fold change between significantly regulated probes. Hybridizations and statistical analysis were performed by the Genomics Facility at Centro Nacional de Biotecnología, CSIC, Spain.

### Microarray data accession number

The microarray data provided in this study have been deposited in NCBI Gene Expression Omnibus [46] genomics data repository and are accessible through GEO Series accession numbers GSE56997 and GSE63728.

### Pyrogallol production by resting cells

To prepare resting cells, cultures of *L. plantarum* WCFS1 were grown in MRS medium at 30 °C to an OD_660_ ≈ 0.3. Cultures were then supplemented with 1.5 mM GA (final concentration) and cultures devoid of GA were used as controls. The GA-induced cultures and their non-induced controls continued to grow at 30 °C to mid-exponential phase (OD_660_ ≈ 0.6) and then centrifuged at 4 °C for 10 min at 3000 rpm. The cells were washed twice with 50 mM potassium phosphate buffer pH 5.8 and resuspended in the same buffer at 4 °C.

The assay for pyrogallol (PG) production was carried out with 1.5 ml of resting cells resuspended to an OD_660_ of 1.5 in 50 mM potassium phosphate buffer pH 5.8. Prior to assays the resting cells were incubated at 30 °C for 10 min. The assay was initiated at time zero with the addition of 1.5 mM GA. Aliquots of 250 µl were removed at fixed time intervals (every 15 or 30 min) and immediately centrifuged for 0.5 min at 14,000 rpm. The supernatant was stored on ice until further analysis by high-performance liquid chromatography (HPLC).

### HPLC analysis

Phenolic compounds were extracted from the supernatants by a standard protocol, involving two extraction steps with one-third of the reaction mixture volume of ethyl acetate. The samples were then analyzed by high-pressure liquid chromatography (HPLC) with a diode array detector. A Thermo chromatograph (Thermo Electron Corporation, Waltham, MA) equipped with a P400 SpectraSystem pump, an AS3000 autosampler, and a UV6000LP photodiode array detector was used. A gradient of solvent A (water and acetic acid, 98:2, vol/vol) and solvent B (water, acetonitrile, and acetic acid, 78:20:2, vol/vol/vol) was applied to a reversed-phase Nova-pack C18 cartridge (25 cm by 4.0 mm [inner diameter]; particle size, 4.6 μm) at room temperature as follows: 0–55 min, 0–80 % solvent B, linear, 1.1 ml/min; 55–57 min, 80–90 % solvent B, linear, 1.2 ml/min; 57–70 min, 90–95 % solvent B, isocratic, 1.2 ml/min; 70–80 min, 95–100 % solvent B, linear, 1.2 ml/min; 80–90 min, 100 % linear, 1.2 ml/min; 100–120 min, washing with methanol 1.0 ml/min; and reequilibration of the column under initial gradient conditions. Detection was performed by scanning from 220 to 380 nm. Samples were injected onto the cartridge in duplicate, after being filtered through a 0.45-μm-pore-size polyvinylidene difluoride filter. The identification of phenolic compounds was carried out by comparing the retention times and spectral data of each peak with those of standards from commercial suppliers. GA and PG concentrations were estimated from the peak areas of the recorded chromatograms according to the “Integration Timed Events and Calibration Finnigan ChromQuestTM 4.2 Data System” software (CHROM-97202; Thermo Electron Corporation-2005).

### Measurement of internal pH (∆pH) and membrane potential (∆Ψ)

The components of the proton motive force were measured as previously described [47]. GA-induced and non-induced resting cells were prepared as described above (when the culture reached an OD_660_ ≈ 0.6) but resuspended at high densities, as outlined below. The internal pH was measured by loading resting cells of *L. plantarum* with the soluble fluorescent pH indicator 2′,7′-bis-(2-carboxyethyl)-5-(and 6)-carboxyfluorescein (BCECF) (Invitrogen Molecular Probes) as previously described [44]. Briefly, 20 µL of resting cells suspended at high density (containing aprox. 50 mg/ml of protein) in 50 mM potassium phosphate (Kpi) buffer pH 5.8 were mixed with 1 µL of a 10 mM BCECF solution and 2.5 µL of 0.5 N HCl and the mixture was incubated 5 min at room temperature to load BCECF. Then the acid shock was stopped by addition of 1 mL of 50 mM Kpi buffer pH 5.8. The BCECF-loaded cells were washed 3 times with 50 mM Kpi buffer pH 5.8, resuspended in 200 µL of the same buffer and kept on ice until use. Fluorescence measurements were performed in 1-cm cuvettes containing 3 mL of 50 mM KPi pH 5.8 buffer equilibrated at 30 °C and 10 µL of BCECF-loaded cells. The mixture in the cuvette was stirred with a magnetic stirring bar. Fluorescence was measured using excitation and emission wavelengths of 502 and 525 nm with slit widths of 4 and 15 nm, respectively. The fluorescence signal was sampled every second in a Fluorolog-3 spectrofluorimeter (Jobin–Yvon-Spex). Opening of the measurement compartment caused loss of data during the first 5–6 s after an addition to the cuvette was made. The internal pH was calculated as described previously [48].

Membrane potential was measured qualitatively with the fluorescent probe 3,3′-dipropylthiocarbocyanine iodide (DiSC_3_) (Invitrogen Molecular Probes) [49]. Increases in electrical potential across the membrane correlate with decreases in fluorescence intensity. A volume of 10 µL of resting cells (OD_660_ ≈ 6 in 50 mM KPi pH 5.8), was added to 3 mL of the same buffer. DiSC_3_ was added from a stock solution to a final concentration of 2 µM to the cuvette containing the cells in 50 mM KPi pH 5.8 buffer. The system was left to equilibrate for 10 min at 30 °C. Fluorescence measurements were performed using excitation and emission wavelengths of 500 and 705 nm, respectively, and slit widths of 8 nm.

## Additional file

10.1186/s12934-015-0345-y *Lactobacillus plantarum* WCFS1 genes with differential expression in presence of gallic acid.
10.1186/s12934-015-0345-y Oligonucleotides used in this study for qRT-PCR analysis.
10.1186/s12934-015-0345-y RT-qPCR validation of nine differentially expressed genes according to microarray data. Linear regression fit analysis was applied and correlation coefficient used for validate the data (y = 0.973x + 0.107; R^2^ = 0.97).
