# Supplementary material for: Molecular adaptation of Lactobacillus plantarum WCFS1 to gallic acid revealed by genome-scale transcriptomic signature and physiological analysis
Source: Microb Cell Fact. 2015 Oct 9;14:160. doi: 10.1186/s12934-015-0345-y (PMC4600210; doi:10.1186/s12934-015-0345-y)
Supplement: Supplementary file 1 — 10.1186/s12934-015-0345-y Lactobacillus plantarum WCFS1 genes with differential expression in presence of gallic acid. [file 12934_2015_345_MOESM1_ESM.pdf]

Additional file 1. Table S1. *Lactobacillus plantarum* WCFS1 genes with differential expression in presence of gallic acid.

Locus Tag	Locus	Description	COG main functional category	Fold Change[a,b]		Subcellular Localization Prediction[c]	Pathway Prediction[c]
				1.5 mM	15 mM		
lp_0226	*nagB*	glucosamine-6-phosphate isomerase/deaminase	Carbohydrate transport and metabolism		2.03	Intracellular	
lp_0250	-	dihidroorotase	General function prediction only	2.06			
lp_0271	*lpdB*	aromatic acid carboxylyase, subunit B	Coenzyme transport and metabolism	19.61	26.30	Intracellular	
lp_0272	*lpdD*	aromatic acid carboxylyase, subunit D		18.84	23.98	Intracellular	
lp_0274	-	TetR family transcriptional regulator	Transcription	4.43	4.15	Intracellular	
lp_0349	*amtB*	ammonium transport protein	Inorganic ion transport and metabolism	-3.14	-7.02	Multi-transmembrane	Sec-(SPI)
lp_0728	*groEL*	GroEL chaperonine	Post-translational modification, protein turnover, and chaperones	-2.14		Intracellular	
lp_0747	*pstD*	phosphate ABC transporter permease	Inorganic ion transport and metabolism		-2.16	Multi-transmembrane	Sec-(SPI)
lp_0748	*pstC*	phosphate ABC transporter permease	Inorganic ion transport and metabolism		-2.16	Multi-transmembrane	Sec-(SPI)
lp_0802	*glnPH1*	glutamine ABC transporter, substrate binding and permease protein	Amino acid transport and metabolism		-3.13	Multi-transmembrane	Sec-(SPI)
lp_0803	*glnQ1*	glutamine ABC transporter, ATP-binding	Amino acid transport and metabolism	-2.26	-2.20	Intracellular	
lp_0822	*glmS1*	glutamine-fructose-6-phosphate transaminase	Cell wall/membrane biogenesis		-3.47	Intracellular	
lp_1061	*rpsK*	30S ribosomal protein S11	Translation		-2.16	Intracellular	
lp_1424	-	oxidoreductase (putative)	General function prediction only		3.44	Intracellular	
lp_1425	-	fumarate reductase, flavoprotein subunit precursor	Energy production and conversion		3.11	Intracellular	
lp_1580	*glnR*	glutamine synthetase repressor	Transcription		-2.10	Intracellular	
lp_1581	*glnA*	glutamate--ammonia ligase	Amino acid transport and metabolism		-2.45	Intracellular	
lp_1590	-	integral membrane protein			2.70	Intracellular/TMH start after 60	Possibly Sec-
lp_1948	-	transcription regulator	Transcription		2.90	Intracellular	
lp_1949	-	integral membrane protein			2.78	Multi-transmembrane	Sec-(SPI)
lp_2105	*cps4D*	UDP N-acetyl glucosamine 4-epimerase, NAD dependent	Multifunctional: Cell envelope biogenesis, outer membrane<br>Carbohydrate transport and metabolism		-2.37	Intracellular	
lp_2229	-	beta-lactamase superfamily metal-dependent hydrolase	General function prediction only		-4.13	Intracellular	
lp_2312	*glnH2*	glutamine/histidine ABC transporter,	Amino acid transport and metabolism		2.34	Lipid anchored	Sec-(SPII)

		substrate binding protein					
lp_2363	*atpC*	H(+)-transporting two-sector ATPase, epsilon subunit	Energy production and conversion		-2.90	Intracellular	
lp_2659	*xpkA*	xylulose-5-P phosphoketolase / fructose-6-P phosphoketolase	Carbohydrate transport and metabolism		-2.35	Intracellular	
lp_2739	-	ABC transporter, ATP-binding protein	Defense mechanisms		-7.25	Intracellular	
lp_2740	-	ABC transporter, permease protein	Secondary metabolites biosynthesis, transport and catabolism		-6.71	Multi-transmembrane	Sec-(SPI)
lp_2741	-	membrane protein			-2.62	Multi-transmembrane	Sec-(SPI)
lp_2742	-	GntR family transcriptional regulator	Transcription		-3.37	Intracellular	
lp_2743	-	ABC transporter ATP-binding protein	Inorganic ion transport and metabolism / Defense mechanisms		-2.67	Intracellular	
lp_2744	-	ABC transporter permease			-2.14	Multi-transmembrane	Sec-(SPI)
lp_2776	*dsdA*	D-serine dehydratase	Amino acid transport and metabolism	-2.08		Intracellular	
lp_2830	*aspA*	aspartate ammonia-lyase	Amino acid transport and metabolism		-2.12	Intracellular	
lp_2940	-	cell surface protein precursor, LPXTG-motif cell wall anchor		10.09	14.33	LPxTG Cell-wall anchored	Sec-(SPI)
lp_2943	-	cation transport protein	Inorganic ion transport and metabolism	51.95	73.41	Multi-transmembrane	Sec-(SPI)
lp_2945	*lpdC*	aromatic acid carboxylyase, subunit C	Coenzyme transport and metabolism	108.47	272.09	Intracellular	
lp_2949	-	integral membrane protein		2.03		Multi-transmembrane	Sec-(SPI)
lp_2954	-	membrane protein			2.92	Multi-transmembrane	Sec-(SPI)
lp_2956	*tanLpI*	tannase (tannin acylhydrolase)	Lipid transport and metabolism	4.24	10.34	Intracellular	
lp_3012	-	oxidoreductase (putative)	Cell wall/membrane/envelope biogenesis / Carbohydrate transport and metabolism	2.49		Intracellular	
lp_3015	-	extracellular protein	Cell wall/membrane biogenesis		-3.43	Secretory(released) (with CS)	Sec-(SPI)
lp_3270	*purA*	adenylosuccinate synthase	Nucleotide transport and metabolism		-2.96	Intracellular	
lp_3271	*guaC*	GMP reductase	Nucleotide transport and metabolism		-3.00	Intracellular	
lp_3659	*rbsD*	D-ribose mutarotase	Carbohydrate transport and metabolism		-2.18	Intracellular	
lp_3660	*rbsK1*	ribokinase	Carbohydrate transport and metabolism		-2.08	Intracellular	

[a] Fold change refers to growth in MRS supplemented with gallic acid 1.5 or 15 mM relative to growth in MRS without supplement

[b] FDR ≤ 0.05; $p<0.05$

[c] LocateP DataBase (http://www.cmbi.ru.nl/locatep-db/cgi-bin/locatepdb.py) CS: CleavageSite; Sec-(SPI): Secretory Pathway I; Sec-(SPII): Secretory Pathway II.
