# Supplementary material for: Molecular adaptation of Lactobacillus plantarum WCFS1 to gallic acid revealed by genome-scale transcriptomic signature and physiological analysis
Source: Microb Cell Fact. 2015 Oct 9;14:160. doi: 10.1186/s12934-015-0345-y (PMC4600210; doi:10.1186/s12934-015-0345-y)
Supplement: Supplementary file 2 — 10.1186/s12934-015-0345-y Oligonucleotides used in this study for qRT-PCR analysis. [file 12934_2015_345_MOESM2_ESM.pdf]

Additional file 2. Table S2. Oligonucleotides used in this study for qRT-PCR analysis.

Locus Tag[a]	Locus	Description	Primer sequence (5′⟶ 3′)
lp_0129	*hsp1*	small heat shock protein	F[b]: TGAGCGTCATACTGGCACCTT<br>R[c]: TCGCCGCAACGTTTGG
lp_0271	*lpdB*	nonoxidative aromatic acid decarboxylase, subunit B	F: GGAGCGTCCGGTACGATTT<br>R: TGGCCGCTGATGTAACTTTTC
lp_0349	*amtB*	ammonium transport protein	F: GGCATGGTCGGCAGTATCAT<br>R: TTGTGCGATTAACGGCTTTG
lp_1036	*rplB*	50S ribosomal protein L2	F: GCAAGGTAAGCGCCCAACT<br>R: CCACCATGAGGGTGATCGTT
lp_1424	-	NADPH-dependent FMN reductase family protein	F: CACTGGTGATGCCAAATATTGAA<br>R: GCCCTGATCATCAAAAGCTTGT
lp_2799	-	amino acid transport protein	F: TCGCAGGTATGGTCGCAAT<br>R: AACAGAATCCCCATGGCAAA
lp_2940	-	cell surface protein precursor, LPXTG-motif cell wall anchor	F: CCTGACCGGTTCGAGTGTTAG<br>R: CATCATGGCCCAGAAAATGAC
lp_2945	*lpdC*	nonoxidative aromatic acid decarboxylase, subunit C	F: GCGGAATCATCCGTTGGA<br>R:ACGAATCGACTTTGGATCATATTG
lp_2956	*tanLp1*	tannase (tannin acylhydrolase)	F: CAACGGCGCCAATTCTG<br>R: GCCGGTCCTGGCAAATAA
**Housekeeping and others internal control genes**			
lp_0006	*gyrB*	DNA gyrase, subunit B	F: CCCGGGTCGCTGCTAAG<br>R: TTTCCAAGCCACTCTTTTTTCG
lp_0007	*gyrA*	DNA gyrase, subunit A	F: CCCGACAGCAACGTCTTCA<br>R: GGCAGCTGGCGTTTGTTT
lp_0789	*gapB*	glyceraldehyde 3-phosphate dehydrogenase	F: CTGGTGCTGCTAAGGCTCTTG<br>R: TGTGCATGGCCTTGTAATTTACC
lp_1021	*rpoB*	DNA-directed RNA polymerase subunit beta	F: GGGTGTGCCTTCTCGTATGAA<br>R: CAGCCATCCCCAAATGCA
lp_1962	*rpoD*	RNA polymerase sigma factor RpoD	F: CGGATCCGCCAAATCG<br>R: CGTGATGGGTGGCGTAACTT
lp_1963	*dnaG*	DNA primase DnaG	F: TCCGGAAGCAGTCGTCAAG<br>R: TCGCCGGCAAGTCAATGT
lp_2057	*ldhD*	D-lactate dehydrogenase	F: AACCGCGACAATGTTTTGATT<br>R: TTGTGAACGGCAGTTTCAGTGT
lp_2301	*recA*	recombinase A	F: CGGCGGGCAGAACAGAT<br>R: GATCCGGACACGGTTACCAA
lp_rRNA01	16srRNA	16S ribosomal rRNA	F: GGGTAATCGGCCACATTGG<br>R: CTGCTGCCTCCCGTAGGA

[a] Designated gene number for the annotated *L. plantarum* WCFS1 genome
[b] Forward
[c] Reverse
