# Supplementary material for: Molecular adaptation of Lactobacillus plantarum WCFS1 to gallic acid revealed by genome-scale transcriptomic signature and physiological analysis
Source: Microb Cell Fact. 2015 Oct 9;14:160. doi: 10.1186/s12934-015-0345-y (PMC4600210; doi:10.1186/s12934-015-0345-y)
Supplement: Supplementary file 3 — 10.1186/s12934-015-0345-y RT-qPCR validation of nine differentially expressed genes according to microarray data. Linear regression fit analysis was applied and correlation coefficient used for validate the data (y = 0.973x + 0.107; R2 = 0.97). [file 12934_2015_345_MOESM3_ESM.pdf]

**Additional file 3. Table S3**. RT-qPCR validation of nine differentially expressed genes according to microarray data. Linear regression fit analysis was applied and correlation coefficient used for validate the data ($y = 0.973x + 0.107$; $R^2 = 0.97$)

| **Locus Tag** | **Locus** | **Description** | **Expression ratios[a, b]** | |
|---|---|---|---|---|
| | | | Microarray[c] | RT-qPCR[d] |
| *lp_2945* | *lpdC* | nonoxidative aromatic acid decarboxylase, subunit C | 8.09 | 8.58 |
| *lp_0271* | *lpdB* | nonoxidative aromatic acid decarboxylase, subunit B | 4.72 | 4.33 |
| *lp_2940* | - | cell surface protein precursor, LPXTG-motif cell wall anchor | 3.84 | 4.38 |
| *lp_2956* | *tanLp1* | tannase (tannin acylhydrolase) | 3.37 | 2.22 |
| *lp_1424* | - | NADPH-dependent FMN reductase family protein | 1.78 | 1.64 |
| *lp_0349* | *amtB* | ammonium transport protein | -2.81 | -2.84 |
| *lp_0129* | *hsp1* | small heat shock protein | 0.35 | 0.19 |
| *lp_0789* | *gapB* | glyceraldehyde 3-phosphate dehydrogenase | -0.28 | -0.27 |
| *lp_1036* | *rplB* | 50S ribosomal protein L2 | -1.02 | -0.67 |
| *lp_2799* | | amino acid transport protein | -0.21 | -0.17 |
| *lp_2057* | *ldhD* | D-lactate dehydrogenase | -1.15 | 0.01 |

[a] Genes ratios >1.5 fold changes (either increase or decrease) were statistically significant ($p < 0.05$); except for *ldhD* gene (regarded as a constitutive gene)
[b] values close to zero indicate no change respect to reference genes (*ldhD*, *16SARNr*, *gyrA*, *dnaG*)
[c] $\log_2$ratio(M), where ratio(M) = fold change
[d] $\log_2$ ratios of average fold change
